# Interprofessional Therapeutic Drug Monitoring of Piperacillin/Tazobactam Enhances Care for Patients with Acute-on-Chronic Liver Failure in the ICU: A Retrospective Observational Pilot Study

**DOI:** 10.3390/antibiotics14020202

**Published:** 2025-02-14

**Authors:** Stephan Schmid, Katharina Zimmermann, Chiara Koch, Patricia Mester, Georgios Athanasoulas, Jonas Buttenschoen, Daniel Fleischmann, Sophie Schlosser-Hupf, Vlad Pavel, Tobias Schilling, Martina Müller, Alexander Kratzer

**Affiliations:** 1 Department of Internal Medicine I, Gastroenterology, Hepatology, Endocrinology, Rheumatology, and Infectious Diseases, University Hospital Regensburg, Franz-Josef-Strauß-Allee 11, 93053 Regensburg, Germany; katharina.zimmermann@ukr.de (K.Z.); chiara.koch@ukr.de (C.K.); patricia.mester-pavel@ukr.de (P.M.); georgios.athanasoulas@ukr.de (G.A.); jonas.buttenschoen@ukr.de (J.B.); sophie.schlosser-hupf@ukr.de (S.S.-H.); vlad.pavel@ukr.de (V.P.); martina.mueller-schilling@ukr.de (M.M.); 2 Hospital Pharmacy, University Hospital Regensburg, 93053 Regensburg, Germany; daniel.fleischmann@ukr.de (D.F.); alexander.kratzer@ukr.de (A.K.); 3 Department of Interdisciplinary Acute, Emergency and Intensive Care Medicine (DIANI), Klinikum Stuttgart, 70174 Stuttgart, Germany; t.schilling@klinikum-stuttgart.de

**Keywords:** therapeutic drug monitoring (TDM), piperacillin/tazobactam, intensive care unit, interprofessional collaboration, acute-on-chronic liver failure

## Abstract

**Background:** Acute-on-chronic liver failure (ACLF) is a severe, rapidly progressing syndrome in patients with liver cirrhosis, often triggered by bacterial infections. Piperacillin/Tazobactam is a key antibiotic in this setting, and therapeutic drug monitoring (TDM) helps optimize its dosing. This study evaluates the impact of an interprofessional TDM strategy for Piperacillin/Tazobactam in ACLF patients in the ICU. **Methods:** This retrospective ICU study evaluated an interprofessional TDM approach for optimizing Piperacillin/Tazobactam dosing in critically ill ACLF patients. The team, consisting of physicians, clinical pharmacists, and staff nurses, engaged in shared decision making, collaboratively interpreting TDM results and adjusting the dosing accordingly. This study included 26 patients with ACLF who underwent initial TDM and 7 who received follow-up TDM. Piperacillin/Tazobactam dosing was modified based on TDM recommendations, with serum concentrations measured weekly. Adherence to and the implementation of interprofessional dosing recommendations were systematically analyzed to assess the impact of this approach. **Results:** The initial TDM showed that 30.8% of patients had Piperacillin/Tazobactam levels within the target range, while 53.8% were above and 15.4% below. The interprofessional team recommended dose reductions in seven patients, increases in three, and no change in eleven, with five requiring antibiotic modifications. At the first follow-up TDM, 20.0% reached target levels, while 80.0% remained above, with no subtherapeutic cases. The team recommended one further dose reduction and maintained dosing in four patients. All recommendations were fully implemented, demonstrating strong adherence to the collaborative protocol. **Conclusions:** The interprofessional TDM strategy optimized Piperacillin/Tazobactam dosing in ACLF patients with full adherence to the recommendations. This collaborative approach improves outcomes and supports global efforts to curb antibiotic resistance.

## 1. Introduction

Acute-on-chronic liver failure (ACLF) is a critical and life-threatening syndrome characterized by acute decompensation in individuals with pre-existing liver cirrhosis, leading to multi-organ failure and high short-term mortality. Without the option of liver transplantation, the prognosis remains poor, with 28-day mortality rates ranging from 18% to 25% for patients with ACLF Grade 1 and escalating to 68–89% for those with ACLF Grade 3 [1]. Timely and comprehensive management at the intensive care unit (ICU) is crucial for improving outcomes in patients with ACLF [2]. Bacterial infections, frequently resulting from bacterial translocation, are among the most common and significant triggers of ACLF, playing a pivotal role in the progression of the disease [3]. This is further exacerbated by the compromised integrity of the mucus layer and the weakened intestinal epithelial barrier commonly observed in patients with cirrhosis, which facilitate bacterial translocation and amplify systemic inflammation [4]. Especially Gram-negative bacteria present a substantial risk [5]. Infections with multidrug-resistant (MDR) bacteria have been rising in recent years, creating significant challenges in treating ACLF patients. These individuals are at high risk for MDR pathogens. This resistance often limits therapeutic options, contributing to worse outcomes in this vulnerable patient population [6].

The European Association for the Study of the Liver (EASL) recommends Piperacillin/Tazobactam as a key antibiotic for treating infections in ACLF patients. These patients are highly susceptible to bacterial infections due to cirrhosis-associated immune dysfunction and frequent bacterial translocation. Prompt and effective antibiotic therapy is essential to prevent infection-related complications that can accelerate organ failure and increase mortality [7,8].

Of note, significant alterations in drug clearance complicate its dosing, as ACLF is often associated with both renal impairments in addition to hepatic dysfunction. While piperacillin is primarily renally excreted, a substantial portion undergoes non-renal clearance, likely through the hepatic metabolism. Impaired liver function, systemic inflammation, and fluid shifts contribute to highly variable serum concentrations, increasing the risk of subtherapeutic levels leading to treatment failure or excessive drug exposure causing toxicity. These challenges necessitate careful dosing adjustments to balance efficacy and safety in critically ill ACLF patients [9,10,11,12].

Therapeutic drug monitoring (TDM) serves as an indispensable tool for optimizing drug dosing in critically ill patients, allowing for individualized adjustments informed by pharmacokinetic and pharmacodynamic parameters [13,14]. In 2020, the Infection Section of the ESICM (European Society of Intensive Care Medicine) highlighted the global significance of TDM in the management of critically ill patients [15]. Within the intensive care unit (ICU), profound alterations in pharmacokinetics, including significant changes in drug clearance and the volume of distribution, are commonly observed [16,17]. These considerations are especially pertinent in patients with ACLF, where extrahepatic organ dysfunction, particularly renal failure, plays a critical role in disease management. Given that most antibiotics, including Piperacillin/Tazobactam, are primarily eliminated via the renal route, the presence of renal impairment necessitates an adjustment of dosing regimens. Furthermore, the use of hemodialysis in these critically ill patients introduces additional complexity to drug clearance, underscoring the importance of precise TDM to maintain therapeutic efficacy while minimizing the risk of toxicity.

Pharmacodynamic alterations in ICU patients establish a link between pharmacokinetic exposure and the antimicrobial’s capacity to either inhibit or eradicate bacterial growth. Recent data have underscored the importance of TDM for antibiotics, specifically in relation to continuous versus intermittent infusion strategies, with Piperacillin/Tazobactam being a particular focus [18,19]. Existing studies have not fully explored the interprofessional management of TDM in patients with severe hepatic dysfunction. This gap is particularly significant for ACLF patients, a high-risk subgroup characterized by complexity and multi-organ involvement.

Beyond the technical aspects of implementing TDM, an interprofessional approach is paramount to ensuring its efficacy and sustainability in clinical practice [20]. This process encompasses the identification of patients requiring TDM, the management of pre-analytical and analytical procedures, and, critically, the interpretation of TDM results to make individualized therapeutic decisions. Effective TDM requires the collaboration of an interprofessional team, including physicians, clinical pharmacists, and nursing staff, each playing a key role in the interpretation and application of TDM findings to optimize patient outcomes.

Interprofessional collaboration and education have become increasingly essential in the context of global healthcare challenges, including workforce shortages, the growing complexity of treatment regimens, an aging population, and the demands imposed by pandemics [21,22]. The World Health Organization (WHO) has recognized interprofessional education and collaboration as strategic priorities for addressing the global healthcare workforce crisis. The integration of interprofessional and interdisciplinary teams within clinical practice not only enhances the precision of therapeutic interventions like TDM, but also supports a holistic approach to patient care, improving outcomes in increasingly complex healthcare environments [23].

The aim of this study was to evaluate the clinical outcomes associated with the implementation of TDM for Piperacillin/Tazobactam in critically ill patients with ACLF managed in the ICU using an innovative interprofessional model. Specifically, this study analyzed the recommendations provided by the interprofessional team and the resultant adjustments to Piperacillin/Tazobactam dosing regimens. To our knowledge, this is the first study to use an interprofessional concept for TDM of Piperacillin/Tazobactam in ICU patients with ACLF. Our study provides a novel approach to optimizing antimicrobial therapy in this critically ill population.

## 2. Results

### 2.1. Demographic and Clinical Characteristics of the Study Cohort

**Figure 1:**
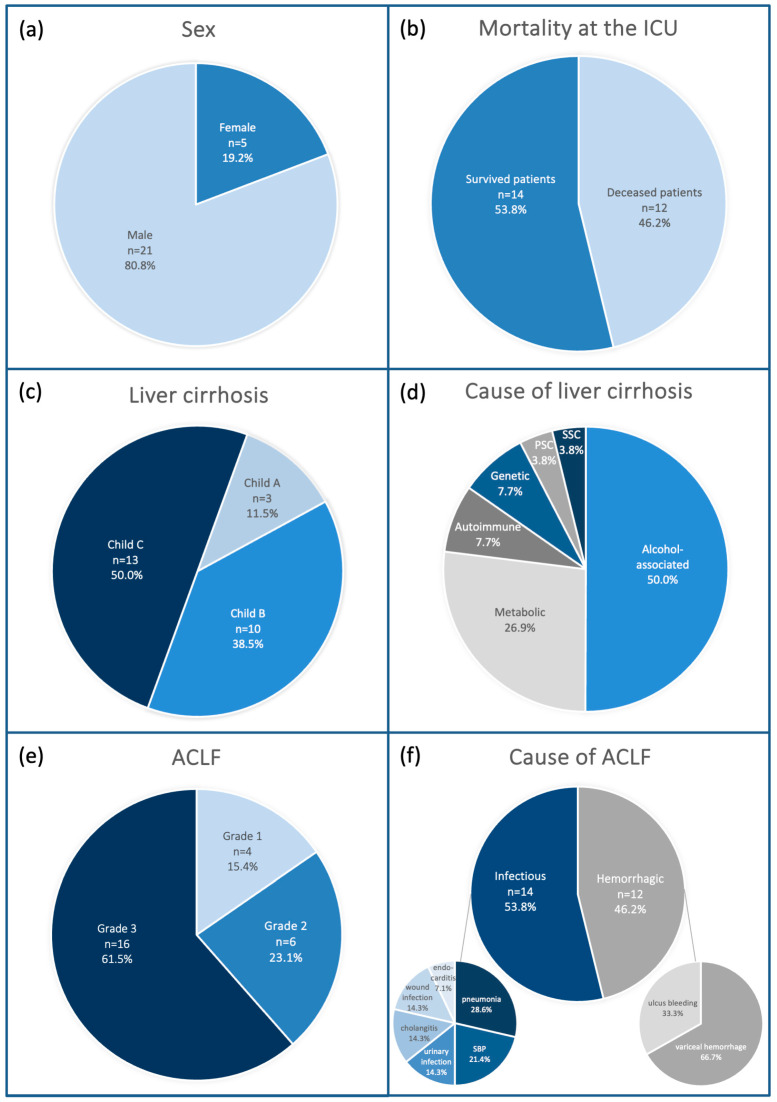
Clinical characteristics of the study population. Twenty-six patients with ACLF were included. All patients were diagnosed with liver cirrhosis and were treated at the ICU due to ACLF. (**a**): Distribution of sex; (**b**): Mortality in the ICU; (**c**): Liver cirrhosis measured by Child–Pugh Score; (**d**): Etiology of liver cirrhosis; (**e**): ACLF grade 1 to 3; (**f**): Causes of ACLF (PSC = primary sclerosing cholangitis, SSC = secondary sclerosing cholangitis SSC).

The demographic and clinical characteristics of the 26 patients included in this study are summarized in Table 1 and Figure 1. The cohort comprised 5 female and 21 male patients, with ages ranging from 26 to 77 years (mean age: 55.1 ± 13.5 years). All patients were admitted to the ICU with a diagnosis of ACLF.

Alcohol-associated liver cirrhosis was the most common underlying etiology of liver disease, identified in 13 patients (50.0%), followed by metabolic dysfunction-associated steatotic liver disease (MASLD) in 7 patients (26.9%), autoimmune liver disease in 2 patients (7.7%), genetic disorders in 2 patients (7.7%), primary sclerosing cholangitis (PSC) in 1 patient (3.8%), and secondary sclerosing cholangitis (SSC) in 1 patient (3.8%). The severity of liver dysfunction was stratified using the Child–Pugh classification: 3 patients (11.5%) were categorized as Child–Pugh A, 10 patients (38.5%) as Child–Pugh B, and 13 patients (50.0%) as Child–Pugh C. The mean MEL score for the cohort was 23.1 ± 7.0, ranging from 10 to 38.

Infection was the primary precipitating factor for acute-on-chronic liver failure (ACLF) in 14 patients (53.8%). The most frequent infections were pneumonia (28.6%), spontaneous bacterial peritonitis (21.4%), urinary tract infections (14.3%), cholangitis (14.3%), wound infections (14.3%), and endocarditis (7.1%). Hemorrhagic events were the triggers for ACLF in 12 patients (46.1%), with the most common causes being esophageal and gastric variceal bleeding (66.7%) and gastro-duodenal ulcer bleeding (33.3%). These patients frequently developed secondary infections, which were treated with Piperacillin/Tazobactam.

All patients included in this study had been previously hospitalized in general wards or other ICUs and had received prior antibiotic therapy. The mean SOFA score was 9.7 ± 2.9 (range: 5–17), indicating a high degree of organ dysfunction. The mean CLIF-C ACLF score was 55.3 ± 11.7 (range: 19–79), reflecting the severity of liver failure and associated complications. Regarding ACLF grading, four patients (15.4%) presented with ACLF Grade 1, six patients (23.1%) with ACLF Grade 2, and sixteen patients (61.5%) with ACLF Grade 3, highlighting the predominance of severe cases in this cohort.

Mechanical ventilation was required in 20 patients (76.9%), reflecting the severity of respiratory dysfunction in this cohort. Renal replacement therapy was administered to five patients (19.2%) due to acute kidney injury or severe metabolic derangements. The mean eGFR of the patients without dialysis was 54.4 (38.1) ml/min/1.73 m^2^.

Overall, 14 patients (53.8%) survived, while 12 patients (46.2%) succumbed to their condition. The primary causes of mortality were septic multiorgan failure, coagulopathy with associated hemorrhage, and refractory circulatory failure, despite maximal intensive care interventions.

This patient cohort was critically ill, exemplifying the severity and complexity of conditions managed in the ICU and underscoring the high mortality rates associated with ACLF. A comprehensive overview of the patient characteristics is presented in Table 1, while Figure 1 highlights the most relevant aspects.

### 2.2. Initial Continuous Piperacillin/Tazobactam Dosing

The initial continuous dosing regimen of Piperacillin/Tazobactam was determined by the ICU physician in accordance with national and international guidelines. Given the pathophysiological alterations in patients with ACLF, particularly the expansion of the third fluid compartment, higher doses of Piperacillin/Tazobactam may be required compared to patients with similar renal function, but without ACLF. In 20 patients (76.9%), the initial dosing of Piperacillin/Tazobactam was set at 500 mg/h (equivalent to 12 g/24 h). In three patients (11.5%), the initial dose was 333 mg/h (8 g/24 h), while one patient (3.8%) received 300 mg/h (7.2 g/24 h), and two patients (7.7%) were administered 250 mg/h (6 g/24 h). In 20 patients (76.9%), Piperacillin/Tazobactam was administered empirically, while in 6 patients (23.1%), the administration was guided by culture results.

### 2.3. Initial TDM Results for Piperacillin/Tazobactam

**Figure 2:**
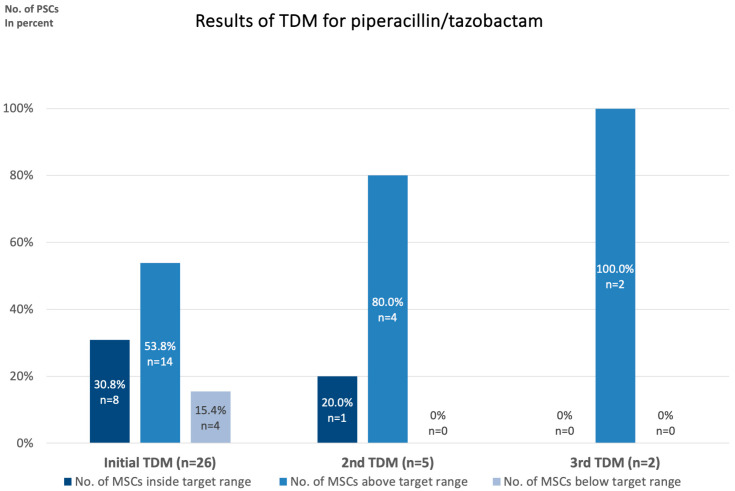
TDM for Piperacillin/Tazobactam with weekly follow-up.

The initial TDM for Piperacillin/Tazobactam revealed a mean serum concentration of 135.8 ± 91.0 (range: 22.8–353.7) across the 26 patients included in the analysis (Table 2, Figure 2). A significant variability in serum concentrations was observed. For the empirical treatment of unidentified bacterial pathogens, a target Piperacillin/Tazobactam serum concentration based on the measured Piperacillin component of 80 mg/L—equivalent to five times the MIC of Pseudomonas aeruginosa, as reported by the European Committee of Antimicrobial Susceptibility Testing (EUCAST)—was pursued [24]. In cases where the causative pathogen was identified, a serum concentration of five times the respective MIC was the therapeutic goal. Bacterial identification was rare in this cohort, likely attributable to prior antibiotic treatments. The identified bacterial species included Klebsiella pneumoniae, Streptococcus agalactiae, and Citrobacter species. The target serum concentration for Piperacillin/Tazobactam was defined as ±25% of the desired concentration, corresponding to a therapeutic range of 60–100 mg/L. According to the literature, a plasma concentration of 157.2 mg/L for Piperacillin was set as the threshold for potential toxicity [25]. A total of eight patients (30.8%) achieved serum concentrations within this target range, whereas fourteen patients (53.8%) had concentrations above the target and four (15.4%) were below the target range. These findings highlight the importance of TDM for Piperacillin/Tazobactam, particularly in the early phases of treatment, to ensure appropriate dosing to prevent resistances and to minimize toxicity. It is very important to note that during the first measurement, eight patients (30.8%) were above the toxicity threshold of 157.2 mg/L.

### 2.4. Follow-Up TDM Results for Piperacillin/Tazobactam

For patients requiring Piperacillin/Tazobactam therapy exceeding seven days, follow-up TDM was performed weekly. In the second week, TDM was conducted in five patients, while in the third week, TDM was conducted in two patients. The mean serum concentration of Piperacillin was 120.4 ± 34.8 mg/L (range: 68.0–165.0) in week 2 and 128.0 ± 9.9 mg/L (range: 121.0–135) in week 3.

In the second week, one patient (20%) had serum concentrations within the target range, with four patients (80%) exceeding this range. By the third week, both patients exhibited serum concentrations above the target range.

Of note, during the 2nd and 3rd measurements, only one patient exceeded the toxicity threshold and no patients had serum concentrations below the target range.

Bar charts show the mean Piperacillin/Tazobactam serum concentration in mg/L with standard deviation at the initial TDM, the second TDM in week 2, and the third TDM in week 3. The initial TDM for Piperacillin/Tazobactam showed a mean serum concentration of 135.8 ± 91.0 (range: 22.8–353.7) mg/L.

### 2.5. Interprofessional Team Recommendations and Implementation

**Figure 3:**
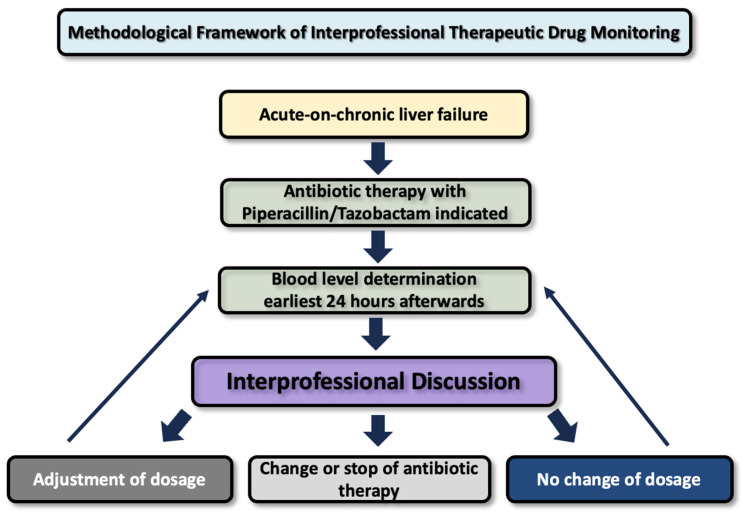
Methodological framework for interprofessional therapeutic drug monitoring.

Serum concentrations of Piperacillin/Tazobactam, determined by HPLC in the hospital’s central laboratory, were reviewed by an interprofessional team. This team included physicians, pharmacists, nurses, medical students, pharmacy trainees, and nurse practitioner trainees. The findings were discussed during interprofessional grand rounds on the same day that the results became available. Clinical recommendations were made considering the patient’s overall clinical status and microbiological findings. A flowchart demonstrating the methodological framework of interprofessional therapeutic drug monitoring is shown in Figure 3.

The figure outlines a stepwise process for TDM, starting with a patient assessment and blood level measurement, followed by an interprofessional discussion to decide on a dosage adjustment, therapy modification, or continuation without changes

**Figure 4:**
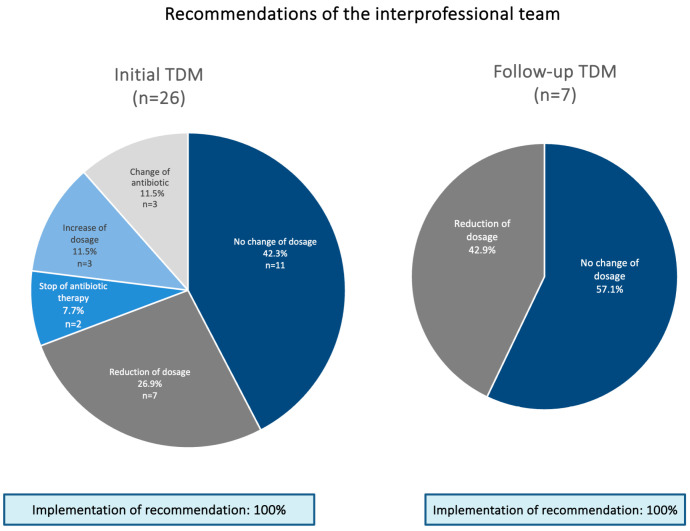
Recommendations of the interprofessional team based on the results of TDM.

Following the initial TDM (therapeutic drug monitoring) assessment, the interprofessional team recommended a dose reduction for seven patients (26.9%), an increase in dosage for three patients (11.5%), and a switch to an alternative antibiotic for three patients (11.5%). For two patients (7.7%), the discontinuation of antibiotic therapy was advised. Only in 11 patients (42.3%) was no change of dosage recommended (Figure 4).

During the second TDM assessment for one patient (20%), a reduction in the dosage was recommended. No adjustments were needed for the remaining four patients (80%).

In the third TDM assessment, a reduction in the dosage was recommended for both patients (100%).

The recommendations differed from maintaining the dose of Piperacillin/Tazobactam, dose adjustments of Piperacillin/Tazobactam, changes in antibiotic therapy, or the complete stop of antibiotic therapy.

## 3. Discussion

This study is the first to systematically evaluate the implementation of an interprofessional approach to TDM in patients with ACLF. The findings highlight the pivotal role of TDM in optimizing Piperacillin/Tazobactam dosing in critically ill patients with ACLF. A significant outcome of this study was the full implementation of all recommendations made by the interprofessional team in 100% of cases. This reflects the feasibility and efficacy of a collaborative, multidisciplinary approach in clinical decision making.

While prior studies have emphasized the importance of TDM in the management of ACLF, this study is unique in investigating the interprofessional dynamics involved and the direct impact on the antibiotic utilization density. These findings underscore the potential of interprofessional TDM strategies to enhance antimicrobial stewardship and improve patient outcomes in this vulnerable patient population.

### 3.1. Cirrhosis-Associated Immune Dysfunction (CAID) and ACLF

Patients with liver cirrhosis demonstrate an increased susceptibility to infections due to cirrhosis-associated immune dysfunction (CAID), which becomes more pronounced as liver disease progresses. Infections are the leading precipitating factor for ACLF, with multidrug-resistant (MDR) bacteria playing an increasingly significant role in its pathogenesis. ACLF is characterized by severe systemic inflammation followed by immune paralysis, creating a challenging clinical scenario that necessitates urgent, individualized management in the ICU [26].

Our findings highlight the importance of incorporating TDM into the care of this vulnerable patient population. Implementing TDM through an interprofessional team approach ensures tailored antimicrobial therapy, addressing both the unique pharmacokinetic challenges in ACLF and the rising threat of MDR infections. This strategy is critical to optimizing treatment outcomes and improving the prognosis for patients with ACLF in the ICU setting.

### 3.2. Continuous Administration of Piperacillin/Tazobactam

In our study, Piperacillin/Tazobactam was administered via continuous infusion during the entire phase of the investigation following an initial loading dose. Continuous administration may enhance the efficacy of Piperacillin/Tazobactam in critically ill patients. Numerous studies have reported higher clinical improvement rates and reduced mortality with continuous administration when compared to intermittent dosing [19,27,28].

Recently, the BLING III Randomized Clinical Trial examined whether the continuous infusion of Piperacillin/Tazobactam or meropenem resulted in decreased all-cause mortality after 90 days in critically ill patients with sepsis compared with intermittent infusion [29]. While no difference in the all-cause mortality could be shown, the clinical cure rate was higher in the continuous infusion group compared to the intermittent infusion group.

### 3.3. Therapeutic Drug Monitoring for Piperacillin/Tazobactam

TDM is essential when administering Piperacillin/Tazobactam via continuous infusion to ensure appropriate dosing, particularly in immunocompromised patients such as those with ACLF, given its bactericidal nature [30,31,32]. In our study, TDM revealed that only 30.8% of initial serum concentrations fell within the target range, while 53.8% exceeded and 15.4% were below the target range. These findings are consistent with the literature, which repeatedly highlights a high rate of failure to achieve target levels for piperacillin in critically ill patients when standard doses are used [33,34,35]. Piperacillin/Tazobactam is predominantly excreted through the kidneys, with 20–32% metabolized by extrarenal pathways. The serum half-life of piperacillin ranges from 0.7 to 1.2 h, with an observed increase of up to 100% in patients with liver cirrhosis [36]. This highlights the need for dosage adjustments based on liver function, which can be evaluated using scores such as the MELD score.

In our interprofessional TDM approach, 53.8% of the initial analyses identified piperacillin overdoses, aligning with findings in the literature, which report excessive drug concentrations above the target range in approximately 60% of patients with liver cirrhosis receiving standard doses [37]. For the beta-lactam antibiotic meropenem, which is similarly primarily renally excreted, Schmid et al. reported an even higher prevalence, with 80% of patients with acute-on-chronic liver failure exhibiting drug concentrations above the target range during the first TDM assessment under standard dosing [38]. In contrast, a study by Schrader et al. demonstrated that in critically ill patients without a specific focus on liver dysfunction, only 26.2% had piperacillin concentrations exceeding the upper target level of 100 mg/L when standard doses were administered [32].

Our study demonstrates that patients with ACLF, often accompanied by impaired renal function, exhibit greater variability in piperacillin serum levels compared to patients with renal failure but without ACLF [10]. While renal impairment is a key determinant of piperacillin clearance, the additional impact of hepatic dysfunction and the frequent volume shifts in ACLF likely contribute to the observed variability. These shifts, driven by systemic inflammation, capillary leakage, and other factors, can significantly alter drug distribution and elimination, further complicating dosing strategies. Our findings underscore the critical importance of therapeutic drug monitoring (TDM), particularly in patients with acute-on-chronic liver failure.

Both underdosing and overdosing pose significant risks. During the initial TDM assessment, the interprofessional team recommended dosage adjustments in 38.5% of cases. Personalized and optimized dosing of Piperacillin/Tazobactam has the potential to markedly reduce the side-effect profile in individual patients, particularly in those with ACLF, who are more susceptible to adverse effects such as seizures and *Clostridioides difficile* infections. Elevated plasma levels of Piperacillin/Tazobactam have been associated with increased neuromuscular excitability and convulsions, particularly when concentrations exceed a value of 157.2 mg/L, which is approximately ten times the MIC for Pseudomonas aeruginosa, exposing patients to unnecessary neurotoxicity without an additional clinical benefit [25].

### 3.4. Interprofessional Collaboration and Shared Decision Making

All recommendations provided by the interprofessional team, comprising physicians, pharmacists, and nurses, were fully implemented in every case. This stands in stark contrast to lower adherence rates reported in previous studies, where the implementation of recommendations has been as low as 50%. The full implementation observed in our study underscores the importance of involving all relevant healthcare professionals in the decision-making process. This inclusive approach supports a collaborative environment, facilitates shared accountability, and ensures collective support for proposed clinical interventions.

Interprofessional education and collaboration are increasingly recognized as essential components in addressing global healthcare challenges and managing the complexities of critical care medicine. In the context of critical care, where patient management often involves multifaceted decisions, interprofessional collaboration plays a pivotal role in optimizing clinical outcomes. In recognition of this, the “I’M A-STAR” initiative was launched in 2020 as a response to the SARS-CoV-2 pandemic. This program was designed to integrate medical, pharmacy, and nurse practitioner trainees into interprofessional collaboration early in their training, providing them with practical exposure to multidisciplinary teamwork.

The “I’M A-STAR” initiative has significantly enhanced the acceptance and awareness of TDM among healthcare teams. This program has not only inspired a culture of interprofessional collaboration, but has also contributed directly to the high rate of the implementation of antibiotic dosing adjustments recommended by the interprofessional team.

### 3.5. Integration of Implementation Frameworks into TDM Practices

The findings of this study, which demonstrate the successful implementation of an interprofessional approach to TDM in Piperacillin/Tazobactam therapy for ACLF patients, align closely with the principles outlined in the Generic Implementation Framework (GIF) proposed by Moullin et al. [39].

As our study highlights, incorporating structured implementation strategies, such as those outlined in the GIF, is critical when introducing and sustaining innovations like TDM in complex clinical environments. By addressing key elements such as context evaluations, influencing factors, and targeted strategies, the GIF provides a valuable blueprint for optimizing the integration of interprofessional TDM practices into routine care.

The full implementation of all interprofessional team recommendations in this study underscores the feasibility and effectiveness of structured approaches in driving change, even in high-stakes settings like the ICU. These insights further emphasize the importance of strategic implementation efforts in addressing the unique pharmacokinetic challenges of ACLF and enhancing the precision of antimicrobial therapy in this vulnerable population.

### 3.6. Lessons Learned

This study highlights the importance of interprofessional TDM in managing Piperacillin/Tazobactam therapy for ACLF patients, addressing the significant pharmacokinetic variability caused by liver dysfunction. The high prevalence of overdosing and underdosing during initial TDM assessments underscores the need for individualized dosing strategies, particularly in critically ill patients.

The full implementation of recommendations by the interprofessional team demonstrates the value of collaborative decision making involving pharmacists, nurses, and physicians. This approach ensures accurate dosing adjustments and fosters a cooperative care environment.

Additionally, combining continuous infusion with TDM proved effective in maintaining therapeutic drug levels, reducing risks of underdosing or overdosing. These findings underscore the potential of interprofessional TDM for antibiotics to optimize outcomes in this vulnerable patient population.

### 3.7. Limitations

This study is a retrospective, single-center analysis with a limited number of included patients, which may limit the generalizability of the findings. The underlying causes of liver cirrhosis in the patient cohort were diverse, and various cofactors could have influenced the results. To enhance the robustness and applicability of these findings, validation through larger, prospective multicenter studies is necessary, allowing for diverse patient populations, standardized protocols, and the consideration of these contributing factors.

## 4. Materials and Methods

### 4.1. Study Design and Patient Characteristics

This retrospective observational cohort study, conducted on an intensive care unit (ICU) of a German university hospital, aimed to evaluate the impact of an interprofessional approach to TDM for Piperacillin/Tazobactam in patients with ACLF in the ICU (inclusion criteria). Exclusion criteria included pregnant patients and individuals who did not consent to participate in studies. Furthermore, moribund patients were excluded from this study. The study period was from January 2023 to September 2023. Data were collected from the patient charts (iMD Soft, Munich, Germany). Ethical approval for this retrospective observational cohort study was obtained from the Ethics Committee of the university hospital (Approval Number: 23-3508-104). Patient consent was waived as this study was conducted as an exclusively retrospective observational analysis, fully compliant with all applicable national and regional regulations to ensure adherence to ethical and legal standards. The study population comprised 26 patients admitted to a medical ICU, which serves as a tertiary referral center for a population of approximately 2.0 million people in southern Germany.

### 4.2. Characteristics of Interprofessional Collaboration and Education

An interprofessional culture has been systematically fostered within the ICU over several years through the implementation of various initiatives, most notably the “I’M A-STAR” project. The “I’M A-STAR” (Intensiv Medizinische AusbildungsSTAtion Regensburg, translating to “Intensive Care Training Ward Regensburg”) initiative, launched in 2020 as a response to the COVID-19 pandemic, established a framework for collaborative, interprofessional training. In this model, medical, pharmacy, and nurse practitioner trainees work as a cohesive interprofessional team at the patient’s bedside, under the close supervision of experienced clinicians, receiving comprehensive interprofessional and interdisciplinary training. This collaborative approach ensures that trainees are actively involved in all aspects of patient care, allowing them to take on significant responsibilities under close supervision, fostering hands-on experience and independent decision making [40]. The ICU’s antibiotic stewardship program [38,41] has also been expanded to include this interprofessional approach to therapeutic drug monitoring for Piperacillin/Tazobactam in ACLF patients.

The interprofessional TDM team consists of specialists in internal medicine, gastroenterology, infectious diseases, and intensive care medicine, as well as clinical pharmacists. The team operates under equal leadership, fostering collaboration and shared decision making among the various disciplines. Beyond a multiprofessional approach, our concept emphasizes a deeply integrated, interprofessional shared decision-making process, ensuring that therapeutic strategies are optimally tailored to individual patient needs [42,43]. All national and international guidelines were carefully reviewed and adhered to, ensuring that the team’s practices align with the highest standards of evidence-based care [44].

Our approach exemplified interprofessional collaboration by emphasizing shared responsibilities and joint decision making among physicians, pharmacists, and nurses. Unlike traditional multidisciplinary frameworks, where contributions from each discipline remain distinct, this approach integrated roles and fostered mutual learning. For instance, while pharmacists typically manage dosing recommendations, nurses and physicians actively participated in the interpretation of TDM results and therapy adjustments. This integrative teamwork created a dynamic, collaborative process, underscoring the educational value of blending expertise across professions [40]. Through this initiative, the ICU continues to focus on the integration of education and clinical practice in a collaborative and interprofessional environment.

### 4.3. Application of Piperacillin/Tazobactam

In the context of ICU management of patients with ACLF, Piperacillin/Tazobactam administration was closely monitored through daily TDM ward rounds involving an interprofessional team, including physicians, pharmacists, nurses, and students. The treatment regimen commenced with an initial loading dose of 4 g/0.5 g of Piperacillin/Tazobactam administered via short infusion, followed by continuous infusion utilizing an automated infusion pump. The Piperacillin/Tazobactam preparation (Piperacillin/Tazobactam Kabi, Fresenius Kabi, Bad Homburg, Germany) involved reconstitution of the lyophilized powder in 50 mL of sodium chloride solution (0.9%, B. Braun Melsungen, Germany) and subsequent transfer to a 50 mL syringe (Original Perfusor^®^ Syringe, B. Braun Melsungen, Germany). A pressure-rated extension set (BD Pressure Rated Extension Sets, BD Switzerland, Eysins, Switzerland) was affixed to the syringe for administration. To maintain the stability of the antibiotic solution, the syringe was replaced every 8 h, ensuring continuous drug delivery throughout the study period, following the initial bolus dose [45].

### 4.4. Therapeutic Drug Monitoring of Piperacillin/Tazobactam

Therapeutic drug monitoring (TDM) was conducted by measuring the total plasma concentration of the piperacillin component. The free (unbound) drug concentration cannot be reliably extrapolated from the total concentration, particularly in the presence of hypoalbuminemia—a condition observed in 96.2% of the patients included in this study [46,47]. Given that previous studies have demonstrated a reduction in piperacillin protein binding to approximately 9% in critically ill patients, compared to 30% in healthy individuals, the protein binding of piperacillin was not considered in this study, consistent with the findings reported in the literature [48].

TDM was conducted on a weekly basis, with blood samples collected every Wednesday at 8:00 AM under steady-state conditions. Steady state was defined as the time elapsed between the initiation of therapy and the first blood sample collection exceeding 24 h. In cases where the duration of Piperacillin/Tazobactam therapy extended beyond seven days, follow-up TDM assessments were performed weekly (i.e., for the second and third TDM), considering the potential of alterations in organ function during the ICU stay. Blood specimens were transported under cooled conditions maintaining a temperature of 2–8 °C to the central laboratory of the hospital, where analysis was performed immediately using high-performance liquid chromatography with ultraviolet detection by a commercially available method-kit (“Antibiotika im Serum/Plasma”—HPLC, Chromsystems Instruments and Chemicals, Gräfelfing, Germany). Upon availability, the TDM results were promptly reviewed and discussed by the interprofessional team. The team provided recommendations regarding any necessary adjustments to the Piperacillin/Tazobactam dosing regimen. Modifications in dosing, decisions to switch to an alternative antibiotic, or discontinuation of antibiotic therapy were implemented by the attending intensive care physician and documented accordingly.

### 4.5. Characterization of Patients with Acute-on-Chronic Liver Failure

The diagnosis of ACLF was made in accordance with the criteria set forth by the European Association for the Study of the Liver—Chronic Liver Failure (EASL-CLIF) consortium guidelines [7].

To assess the severity and prognosis of liver disease, several scoring systems were utilized, including the Child–Pugh score [49], Sequential Organ Failure Assessment (SOFA) score [50], Model for End-Stage Liver Disease (MELD) score [51,52], and CLIF-C ACLF score [53].

The MELD score, which serves as a predictor of mortality prognosis in patients with end-stage liver disease, was calculated using the following formula [51,52]:

MELD score = 9.57 × ln(serum creatinine) + 3.78 ln(total bilirubin) + 11.2 × ln(international normalized ratio) + 6.43.

Additionally, the CLIF-C ACLF score, which further evaluates prognosis in ACLF patients, was determined by the following equation [53]:

CLIF-C ACLF score = 10 × (0.33 × CLIF-C-OFs + 0.04 × Age + 0.63 × ln (WBC count in 10^3^/μL) − 2).

Both scoring systems were applied to assess the clinical characteristics and prognosis of the patient cohort. This structured evaluation provides critical insights into the extent of organ dysfunction and overall prognosis in patients with ACLF during ICU admission.

### 4.6. Statistical Analyses and Data Collection

Primary data were retrieved from the hospital’s electronic health record systems, including SAP^®^ (SAP SE, Walldorf, Germany) and the Metavision^®^ patient data management system (Meona GmbH, Freiburg, Germany). Pharmacoeconomic data were supplied by the hospital’s pharmacy department. Statistical analyses were conducted using SPSS^®^ Version 28.0.0 (Statistical Package for the Social Sciences, IBM Corporation, Armonk, New York, NY, USA). For hypothesis testing, one-tailed t-tests were applied, with statistical significance set at a *p*-value of ≤0.05.

## 5. Conclusions

This study demonstrated that the interprofessional approach to TDM for Piperacillin/Tazobactam not only facilitated the optimization of dosing regimens, but also achieved 100% adherence to the recommendations provided by the interprofessional team, thereby enhancing both patient safety and therapeutic efficacy. The complete implementation of the recommendations underscores the critical value of collaborative decision making among healthcare professionals in ensuring the precise and effective application of antimicrobial therapy.

The rational and judicious use of antibiotics such as Piperacillin/Tazobactam is important, not only for improving individual patient outcomes, but also for addressing broader global health concerns, including antimicrobial resistance. By optimizing dosing strategies, TDM contributes to minimizing unnecessary drug exposure and reducing the selection pressure for resistant organisms, thereby supporting global efforts in antimicrobial stewardship.

## Figures and Tables

**Table 1:** Clinical characteristics of the study population.

Characteristics	Total Study Population (*n* = 26)
Age [years]: mean ± SD (range)	55.1 ± 13.5 (26–77)
Sex: *n* (%)	
Female	5 (19.2)
Male	21 (80.8)
SOFA Score [points]: mean ± SD (range)	9.7 ± 2.9 (5–17)
Dialysis: *n* (%)	5 (19.2%)
eGFR [ml/min/1.73 m^2^]: mean ± SD (range)	54.4 (38.1)
Mortality at the ICU: *n* (%)	
Deceased patients	12 (46.2)
Survived patients	14 (53.8)
Liver cirrhosis: *n* (%)	26 (100.0)
Child A/B/C	3 (11.5)/10 (38.5)/13 (50.0)
Cause of liver cirrhosis: *n* (%)	
Alcohol-associated	13 (50.0)
Metabolic	7 (26.9)
Autoimmune	2 (7.7)
Genetic	2 (7.7)
Primary sclerosing cholangitis	1 (3.8)
Secondary sclerosing cholangitis	1 (3.8)
MELD score [points]: mean ± SD (range)	23.1 ± 7.0 (10–38)
ACLF: *n* (%)	26 (100.0)
ACLF Grade 1/2/3	4 (15.4)/6 (23.1)/16 (61.5)
Cause of ACLF: *n* (%)	
Infectious	14 (53.8)
pneumonia	4 (28.6)
spontaneous bacterial peritonitis	3 (21.4)
urinary infection	2 (14.3)
cholangitis	2 (14.3)
wound infection	2 (14.3)
endocarditis	1 (7.1)
hemorrhagic	12 (46.1)
variceal hemorrhage	8 (66.7)
ulcus bleeding	4 (33.3)
CLIF-C-ACLF-Score [points]: mean ± SD (range)	55.3 ± 11.7 (19–79)
Initial continuous dosing of Piperacillin/Tazobactam [mg/h]: mean ± SD (range)	
	453.8 ± 87.9 (250–500)

Presentation of the baseline demographic (age, sex) and clinical (ICU stay, mortality in the ICU, liver cirrhosis, cause of liver cirrhosis, MELD score, ACLF, cause of ACLF, and CLIF-C-ACLF score) characteristics of the total study population and of the initial continuous dosing of piperacillin/tazobactam.

**Table 2:** Results of TDM for Piperacillin/Tazobactam.

Piperacillin Serum Concentration (PSC)	Initial TDM (n = 27)	2nd TDM (n = 5)	3rd TDM (n = 2)
PSC [mg/L]: mean ± SD (range)	135.8 ± 91.0 (22.8–353.7)	120.4 ± 34.8 (68.0–165.0)	128.0 ± 9.9 (121.0–135)
No. of PSCs inside target range (%)	8 (30.8)	1 (20.0)	0
No. of PSCs above target range (%)	14 (53.8)	4 (80.0)	2 (100)
No. of PSCs below target range (%)	4 (15.4)	0 (0.0)	0 (0.0)

Presentation of the Piperacillin serum concentrations (PSC) collected during initial, 2nd, and 3rd TDM in our total study population.

## Data Availability

The raw data supporting the conclusions of this article will be made available by the authors, without undue reservation.

## Abbreviations

ABS	Antibiotic stewardship
ACLF	Acute-on-chronic liver failure
A-STAR	AusbildungsSTAtion Regensburg = German for “interprofessional training ward Regensburg”
CAID	Cirrhosis-associated immune dysfunction
CDI	*Clostridioides difficile* infection
CLI	Chronic liver injury
CLIF-C	Chronic Liver Failure—Consortium
EASL-CLIF	European Association for the Study of the Liver— Chronic Liver Failure
ESICM	European Society of Intensive Care Medicine
GIF	Generic Implementation Framework
HPLC	High-performance liquid chromatography
ICU	Intensive care unit
I’M A-STAR	IntensivMedizinische AusbildungsSTAtion Regensburg = German for “Intensive care training ward Regensburg”
MDR	Multidrug-resistant
MELD	Model for End-Stage Liver Disease
MIC	Minimum inhibitory concentration
MSC	Meropenem serum concentrations
PD	Patient day
RDD	Recommended daily doses
SD	Standard deviation
TDM	Therapeutic drug monitoring
WHO	World Health Organization
